# Bone Scintigraphy of Vertebral Fractures With a Whole-Body CZT Camera in a PET-Like Utilization

**DOI:** 10.3389/fnume.2021.740275

**Published:** 2021-09-10

**Authors:** Achraf Bahloul, Antoine Verger, Alain Blum, Mohammad Bilal Chawki, Mathieu Perrin, Saifeddine Melki, Gilles Karcher, Pierre-Yves Marie, Laetitia Imbert

**Affiliations:** ^1^Université de Lorraine, CHRU-Nancy, Department of Nuclear Medicine and Nancyclotep Imaging Platform, Nancy, France; ^2^Université de Lorraine, INSERM U1254, IADI, Nancy, France; ^3^Université de Lorraine, CHRU-Nancy, Department of Radiology Guilloz, Nancy, France; ^4^Université de Lorraine, INSERM, UMR-1116 DCAC, Nancy, France

**Keywords:** CZT, SPECT, bone scintigraphy, vertebral fracture, standard uptake value

## Abstract

**Objective:** An image display with a standardized uptake value (SUV) scale is recommended for analyzing PET exams, thus requiring the reconstruction of accurate images for both SUV measurement and visual analysis. This study aimed to determine whether such images may also be obtained with a high-speed CZT-SPECT/CT system, with a further application for the longitudinal monitoring of vertebral fractures.

**Materials and Methods:** SPECT image reconstruction was optimized with an IEC phantom according to both image quality parameters and accuracy of measured activity. The optimized reconstruction process was applied to ≤15 min ^99m^Tc-HDP SPECT spine recordings previously acquired from 25 patients (74 ± 12 years old) at both early (1.3 ± 1.1 months) and late (5.2 ± 2.3 months) stages after an acute vertebral fracture.

**Results:** A SPECT reconstruction with 32 equivalent iterations was selected based on the association of high detectability for spheres down to 0.6 ml in volume, with accurate measured activity, although the latter was affected by partial volume effect for spheres ≤5.6 ml. Coherent measurements were obtained on these high-quality SPECT images for the SUVmax from the intact vertebrae of patients, which were stable between basal SPECT/CT and follow-up SPECT/CT (for T1 vertebrae: 5.7 ± 1.1 vs. 5.8 ± 1.1, *p* = 0.76), and from initially fractured vertebrae, which were dramatically higher on the basal compared with the follow-up SPECT (21.0 ± 8.5 vs. 11.2 ± 4.2, *p* < 0.001), whereas inverse changes in SUVmax were observed for newly compacted fractures identified on follow-up SPECT (74.4 ± 2.0 vs. 21.8 ± 10.3, *p* = 0.002). Finally, an image display with an SUV scale was shown to be advantageous for highlighting areas with >7.5 SUV, a level reached by 98% of vertebral fractures of <7 months and 4% of reference intact vertebrae.

**Conclusion:** Bone scintigraphy of vertebral fractures may be obtained with this CZT-SPECT/CT system with fast 3D acquisitions and high-quality images displayed with a reliable SUV scale, approaching what is achieved and recommended for PET imaging.

## Introduction

An image display with a fixed standardized uptake value (SUV) scale is recommended for analyzing PET exams, likely enhancing the robustness and reproducibility of image analysis (1, 2). This requires the reconstruction of accurate images for both SUV measurement and visual analysis, and unfortunately, this goal is more difficult to achieve with conventional SPECT imaging.

However, several recently published studies have provided evidence of a significant contribution of absolute quantification in the interpretation of bone SPECT images, especially for the monitoring of longitudinal changes after treatment (3–5) and also for the characterization of metastasis (6–8), osteomyelitis, osteonecrosis (9), and certain degenerative bone lesions (10, 11). All these previous quantitative bone SPECT studies were conducted with conventional Anger cameras, and none has presented an image display using an SUV scale and with whole-body recording times adapted to routine clinical use. These limitations could be overcome with a new high-speed whole-body CZT camera, which has the advantages of not only enhancing image quality but also reducing SPECT recording times, thus enabling a whole-body acquisition in <20 min (12, 13), similar to recording times currently achieved for PET imaging.

In particular, the whole-body VERITON^®^ camera (Spectrum Dynamics Medical, Caesarea, Israel) combines CZT detectors with an original 360° ring-configuration geometry that is likely to maximize both count sensitivity and image quality (12–14). Absolute quantification has also been developed with this camera and applied on lutetium-177 imaging (13), although as yet there has not been any assessment for routine bone scintigraphy.

The present study aimed to determine whether the high-speed VERITON SPECT/CT system may provide images for both reliable SUV measurement and high image quality—that is, the conditions required to enable visual analysis of images displayed using an SUV scale—with a further application for the longitudinal monitoring of vertebral fractures.

## Materials and Methods

All images were acquired on the VERITON^®^ hybrid system comprising a high-sensitivity 360° CZT camera and 64-row detector CT. The present study involves the analysis of a routine protocol for bone SPECT/CT recording applied (i) on a phantom for optimizing the image reconstruction process, with respect to image quality and absolute quantification measurement, and (ii) in patients with acutely fractured vertebrae to check for the consistency of SUV measurement, and especially for changes over time in fractured vs. non-fractured vertebrae.

The study was approved on January 22, 2021, by the Ethics Committee of the CHRU of Nancy (reference number 297). This research complied with the principles of the Declaration of Helsinki. Informed consent was obtained from all individuals included in the study.

### Bone SPECT/CT Recordings of Patients

Twenty-five consecutive patients were retrospectively selected on the basis of prior imaging on the VERITON^®^ hybrid system, comprising a basal SPECT/CT for pretherapeutic assessment prior to a possible vertebral augmentation procedure after an acute vertebral fracture of traumatic and/or osteoporotic origin and a follow-up SPECT/CT at a later stage, mainly due to suspicion of new vertebral fractures (vertebral fracture cascade). The fracture date was considered to be that of the acute onset or worsening of pain.

SPECT/CT acquisitions commenced 3 to 4 h after the injection of approximately 550 MBq of ^99m^Tc-hydroxydiphosphonate (HDP). CT was recorded first, with the following parameters: 120 kV, 150 mA s modulation, pitch of 0.8, slice thickness of 1.25 mm, increment 1.25 mm, iterative reconstruction, and bone filter. SPECT was recorded thereafter with the detectors placed as close as possible to the patients, with an energy window of 140 keV ± 7.5%, two to three bed positions of 5 min recording each, and a number of projections ranging from 240 to 360 for each detector (depending of the imaged object size).

### Phantom Experiments

The CZT camera was previously calibrated for clinical routine reconstruction parameters using a cylinder filled in with a homogeneous solution of ^99m^Tc, as previously described (15).

A NEMA IEC body phantom (Data Spectrum Corporation, Durham, NC, USA) was filled in within the background compartment using a ^99m^Tc solution with an activity concentration of 16.50 kBq ml^−1^ at scan time and with an 8.3-fold higher activity concentration (137.05 kBq ml^−1^) within six spherical inserts having respective volumes of 0.6, 1.1, 2.6, 5.6, 11.5, and 26.5 ml. This phantom was recorded at the center of the camera field of view using the routine protocol recommended for bone scintigraphy, although with recording time reduced to 2 min in a single bed position. Accordingly, the total number of recorded counts was in the range observed for the thoraco-abdominal bed positions of our routine bone scintigraphy recordings (1.7 Mcounts).

### Image Reconstructions and Analysis

SPECT images were reconstructed using an ordered subset expectation maximization (OSEM) iterative method recommended for routine bone SPECT with this camera, after initially testing a range of 2 to 10 iterations for the reconstruction of the IEC phantom images. The remaining parameters were fixed as follows: 8 subsets, kernel inter-iteration filter with a factor 0.2, postreconstruction median filter (3 × 3 × 3 voxels), and several corrections were applied with the point-spread function (PSF) method including scatter correction, resolution recovery, attenuation correction with the CT attenuation map, and partial volume correction with a dedicated vendor-proprietary algorithm (PVC) that uses the anatomical information provided by the CT scan. For the phantom images, this PVC algorithm was applied on a CT scan for which the density of the spheres was fixed at 700 Hounsfield units (HU), that is, an intermediate level between the HU from spongious and cortical bone. All SPECT images were displayed through cubic voxels of 2.46 × 2.46 × 2.46 mm^3^.

The MIM software (MIM Software Inc., Cleveland, OH, USA) was used to provide paired display of SPECT and CT images. Volumes-of-interest (VOIs) were drawn manually, encompassing (1) each of the six spheres of the IEC phantom and (2) the bodies of targeted fractured vertebrae and the reference T1 vertebra on SPECT/CT exam of each patient. The targeted fractured vertebrae were defined as those showing the highest SUVs on SPECT recordings, together with typical CT signs of bone compression and/or fracture. In addition, the T1 vertebra was used as a control reference since this vertebra is at very low risk of osteoporotic fractures (16), and no fracture or severe arthrosis lesion was detected on T1 vertebra on any of our SPECT/CT recordings. Additionally, cement volume was excluded from the VOIs of all vertebrae treated by cementoplasty.

Mean HU were obtained from the CT vertebral VOIs; maximal SUV (SUVmax) from the SPECT vertebral VOIs and a maximal activity concentration expressed in % of the actual concentrations were computed with the SPECT phantom VOIs. SUVs were determined with the following formula: *A*_VOI_ × *BW*/*A*_INJ_, where *A*_VOI_ is the activity concentration within a VOI in kBq/ml, *A*_INJ_ is the decay-corrected injected activity in kBq, and *BW* is the patient body weight in g.

A contrast-to-noise ratio (C/N) was computed on each SPECT image reconstruction of the IEC phantom, with a spherical VOI of 18 mm diameter corresponding to half the diameter of the largest sphere. This VOI was placed at the center of the largest sphere to measure mean sphere activity (“Mean sphere” in counts per second) and corresponding standard deviation (“SD sphere”). A 5-cm translation of the VOI was subsequently applied to measure mean background activity (“Mean BKG” in counts per second) and corresponding standard deviation (“SD BKG”). Contrast-to-noise ratio was finally computed with the following equation (17): $\frac{Meansphere-MeanBKG}{\sqrt{SDsphere^{2}+SDBKG^{2}}}$.

### Statistical Analysis

Qualitative variables were expressed with frequencies and quantitative values, with means ± standard deviations. Mann–Whitney tests and Wilcoxon signed-rank tests were used, respectively, for the unpaired and paired comparisons of quantitative variables. For all tests, a *p*-value <0.05 was considered as indicative of a significant difference. This statistical analysis was performed with SPSS 25.0 software (IBM Corp^®^, Armonk, NY, USA).

## Results

### SUV Measurement and Image Quality According to Reconstruction Parameters

**Figure 1:**
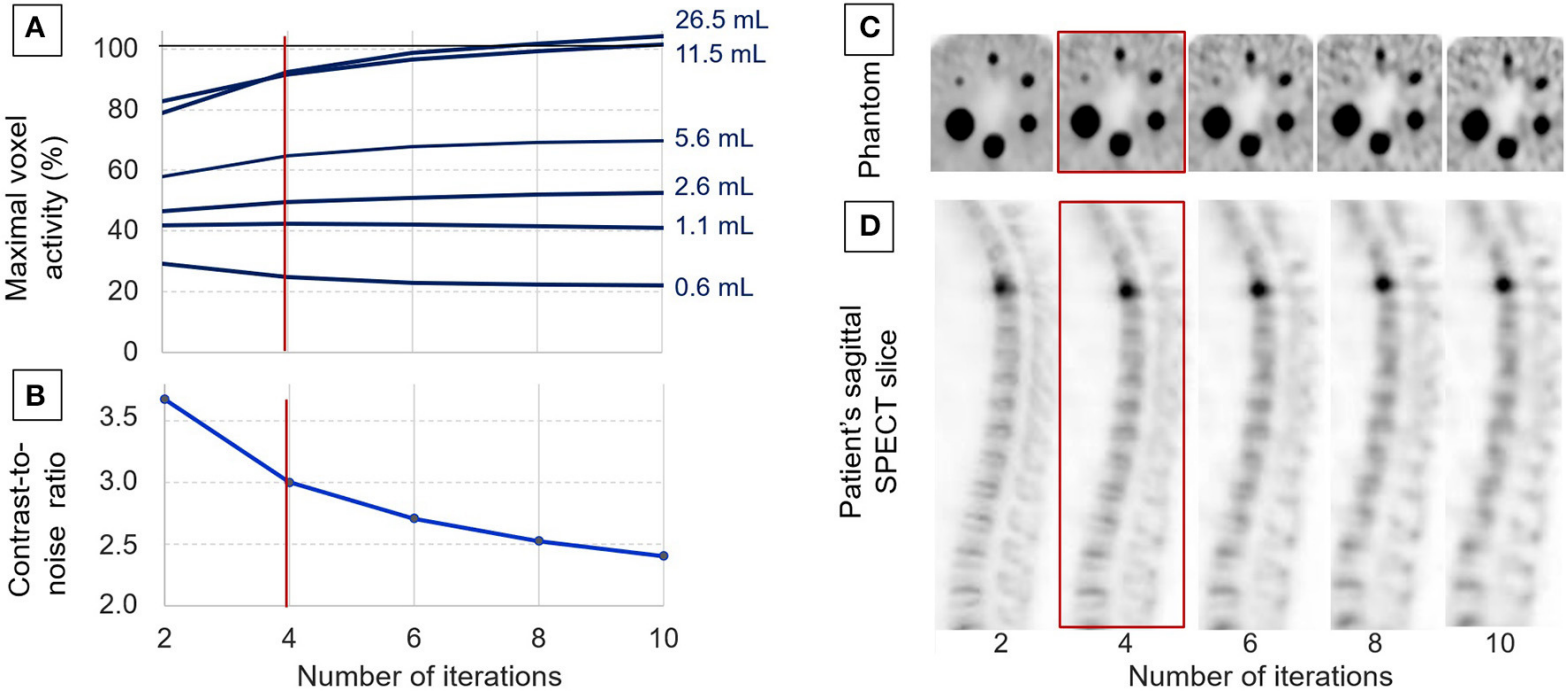
Data used for adjusting the OSEM reconstruction process with analyses of the evolutions, according to the number of iterations: of the maximal voxel activity concentration measured on the spheres of the IEC phantom and expressed relative to the actual activity concentration **(A)**, the evolution of the contrast/noise ratio determined on the IEC phantom **(B)**, representative tomographic SPECT slices from the IEC phantom **(C)**, and from a patient in a sagittal orientation passing through a fractured T4 vertebra **(D)**. Four iterations were the number considered to provide the best compromise between image quality and the accuracy of activity measurements, and it was thus selected for further clinical applications.

As detailed in Figure 1, the SUVmax measured on most phantom spheres exhibited an increase according to the number of iterations used in the OSEM reconstruction process. The actual activity concentration level was reached with eight or more iterations, but only for the two largest spheres of 26.5 and 11.5 ml. Notably, more than 90% of the actual level was reached with only four iterations for these two largest spheres. A significant underestimation was then documented for activities in sphere volumes of 5.6 ml or lower (Figure 1).

As evidenced in the curves and images from Figure 1, the contrast-to-noise ratio exhibited a marked decrease when increasing the number of iterations, and this was associated with a significant deterioration in the detectability of the smallest spheres and bone structures.

Finally, the reconstruction process with four iterations (i.e., 32 OSEM equivalent iterations and a kernel inter-iteration filter with a factor of 0.2) was selected for additional further analyses, based on high levels of (i) contrast/noise ratio, (ii) detectability for spheres down to a 0.6-ml volume, and (iii) accuracy of activity measurements, although this was affected by partial volume effect for spheres ≤5.6 ml, as already stated above (Figure 1).

### Characteristics of the Study Population

At the time of the basal SPECT/CT, the mean age of the study patients was 73 ± 12 years (51 to 90 years), 11 (44%) were women, and 15 (60%) had a previous history of vertebral compression fracture. An at least partially osteoporotic mechanism was suspected for all these acute fractures except in four patients for whom this mechanism was presumed purely traumatic.

The basal SPECT/CT was performed at a mean of 1.3 ± 1.1 months after the acute fracture date. It was prescribed as part of a pretherapeutic evaluation, based on which 12 patients were referred for cementoplasty and 13 referred for conservative treatment involving a back brace.

The follow-up SPECT/CT was performed at a mean of 5.2 ± 2.3 months after the initial acute fracture episode and was prescribed for the persistence or resurgence of back pain in all patients. A new episode of acute vertebral fracture was finally diagnosed in 12 patients for whom the mean delay time between symptom resurgence or aggravation and follow-up was 4.9 ± 4.1 weeks.

Overall, a total of 37 vertebrae were considered as acutely fractured, based on evocative symptoms and analysis of both the basal and follow-up SPECT/CT. In order of decreasing frequency, these fractures were located on L2 in seven cases; L1 and T12 in five cases each; T11 and L4 in four cases each; T7 in three cases; T4, T10, and L3 in two cases each; and T5, T6, and L5 in one case each.

### Results of SPECT/CT Imaging and Image Display With the SUV Scale

As detailed in Table 1, SUVmax measurements from the reference intact T1 vertebrae remained stable between the basal and follow-up SPECT/CT (5.7 ± 1.1 vs. 5.8 ± 1.1, *p* = 0.76). By contrast, SUVmax from the initially fractured vertebrae was dramatically higher on the basal than on the follow-up SPECT (21.0 ± 8.5 vs. 11.2 ± 4.2, *p* < 0.001), whereas inverse changes were documented for newly compacted fractures apparent on the follow-up SPECT (7.4 ± 2.0 vs. 21.8 ± 10.3, *p* = 0.002). These latter differences were associated with an increase in bone density, between the basal and the follow-up SPECT/CT (121 ± 28 vs. 190 ± 39 HU, *p* = 0.003) (Table 1).

**Table 1:** Paired comparisons between the basal and follow-up SPECT/CT of imaging parameters recorded on 25 initially fractured vertebrae and 25 reference non-fractured T1 vertebrae, as well as for 12 vertebrae identified as newly fractured on the follow-up SPECT.

	**Basal SPECT/CT**	**Follow-up SPECT/CT**	***P*** **-value**
**Reference T1 vertebrae (*****n*** **= 25)**			
SUVmax	5.7 ± 1.1	5.8 ± 1.1	0.76
Mean HU	178 ± 32	173 ± 31	0.22
**Initially fractured vertebrae (*****n*** = 25^*^)			
SUVmax	21.0 ± 8.5	11.2 ± 4.2	<0.001
Mean HU	232 ± 54	232 ± 59	0.71
**Newly fractured vertebrae (*****n*** = 12^*^)			
SUVmax	7.4 ± 2.0	21.8 ± 10.3	0.002
Mean HU	121 ± 28	190 ± 39	0.003

* *Only vertebrae showing the highest SUVmax on the spine SPECT images were selected for these analyses, based on the assumption that they are those corresponding to the recent episode of occurrence or recurrence of pain*.

**Figure 2:**
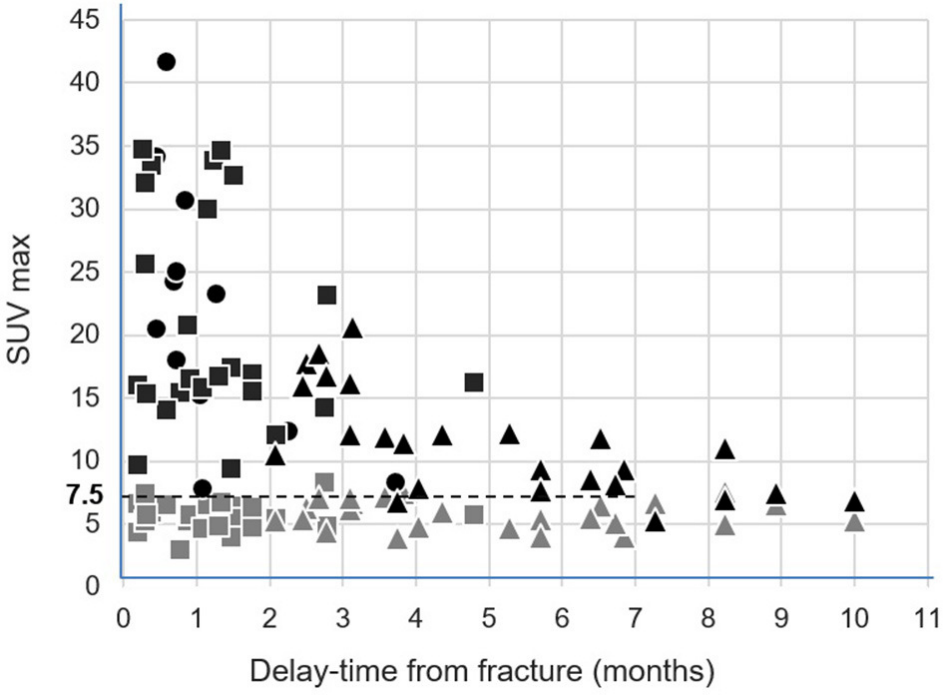
Representations of the SUVmax from the 25 intact control T1 vertebrae (gray symbols) and from the 25 initially fractured vertebrae (black symbols), measured on the basal SPECT/CT (rectangle) and follow-up SPECT/CT (triangle), according to delay time from the fracture date. The SUVmax from the 12 newly fractured vertebrae apparent on the follow-up SPECT/CT were additionally inserted according to delay time from the corresponding fracture date (black circles).

All SUVmax values measured on T1 and on fractured vertebrae are displayed according to the delay time from the fracture date in Figure 2. This figure shows a decline over time in the SUVmax from fractured vertebrae. On a receiver-operating characteristic (ROC) curve analysis, the threshold of an SUVmax >7.5 was the best criterion for differentiating the fractured vertebrae from the intact T1 vertebrae. This discrimination was even better when only considering the vertebrae fractured in the preceding 7 months, as evidenced in Figure 2. As many as 98% (56/57) of these fractured vertebrae had an SUVmax >7.5, whereas this rate was only 4% for reference intact T1 vertebrae (2/50).

**Figure 3:**
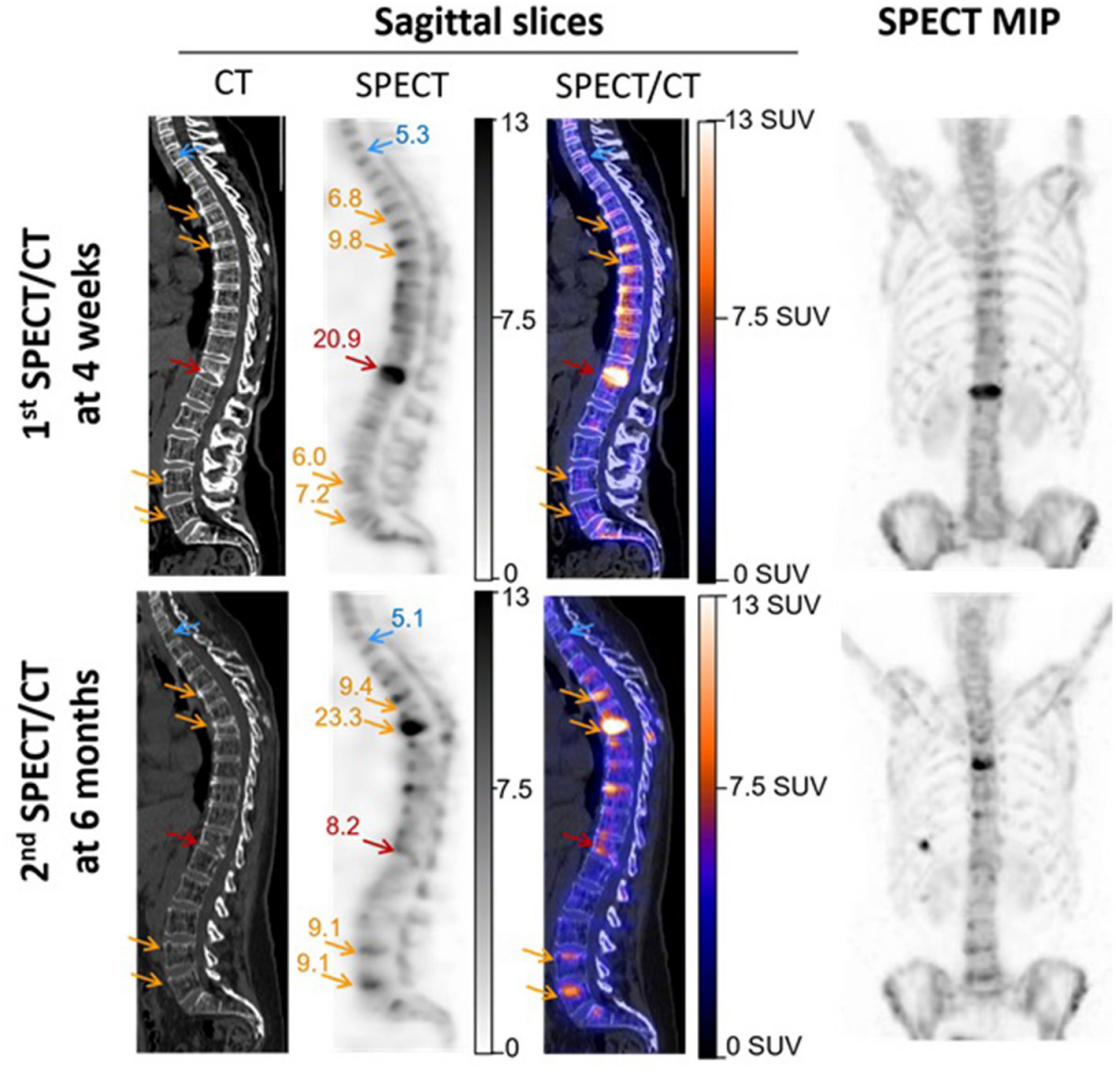
SPECT/CT images recorded in a 56-year-old woman with severe osteoporosis, 4 weeks after a T12 fracture, and additionally 6 months later, for suspected additional fractures. These latter were finally confirmed on T5, T7, L4, and L5 in a framework of cascade fractures. Images are displayed through sagittal SPECT, CT, and fused SPECT/CT images and anterior projections of the maximal intensity projection (MIP) of SPECT images. All SPECT images are similarly scaled from 0 to 13 SUV, thereby facilitating the observations of (i) SUVmax stability between the two time points for the reference non-fractured T1 vertebra (blue arrows), and by contrast, (ii) a significant increase (orange arrows) or decrease (red arrows) in the SUVmax for fractured vertebrae. The SUVmax levels are inserted in the SPECT images, and it may be noted that all the indicated bone areas with increased SUVmax (orange arrows) are associated with concomitant changes in vertebra shape on CT, confirming more or less severe compressions.

These results are illustrated in Figure 3 by SUV-scaled images from the same patient with scales adjusted to a maximum set at 13 SUV for both the basal and follow-up SPECT. This likely leads to facilitate the comparison between the two SPECTs and to favor the identification of bone structures reaching a ≥7.5 SUV level (i.e., orange for the color scale and dark gray for the gray scale). In particular, the initially fractured vertebra remains detectable on the follow-up SPECT, despite a marked decreased uptake from the basal SPECT. Opposingly, an increased uptake may be easily detected between the basal and follow-up SPECT on several vertebrae showing CT signs of new compressions on the follow-up SPECT/CT, in a typical setting of vertebral cascade fracture.

## Discussion

This study shows that in conditions of image reconstruction targeting a high level of image quality, high-speed recording from this whole-body CZT-SPECT/CT system provides reliable and coherent SUVmax measurements in patients with vertebral fractures and/or compressions. These properties were required to enable a visual analysis of images displayed using the SUV scale, as already achieved and recommended for enhancing the robustness and reproducibility of PET analyses (1, 2).

Overall, our phantom results agree with the conclusions achieved by previous studies planned on conventional Anger cameras, regarding the acceptable accuracy of SPECT SUV measurements (18, 19), especially for volumes ≥10 ml (20) and when associated with CT-based methods enabling reduction in the partial volume effect (21–23). It is notable, however, that underestimation of SUVmax is less marked when using an alternative reconstruction method available from the manufacturer and which favors spatial resolution to a greater extent (i.e., 64 OSEM equivalent iterations and a kernel inter-iteration filter with a factor of only 0.125) (see Supplementary Figure). This would be a better choice for further enhancing the precision of SUV determination, such as required for a dosimetric estimation (13), but at the expense of a lower contrast/noise ratio for the visual analysis of images displayed with an SUV scale (see Supplementary Figure).

Therefore, the reconstruction parameters applied in the patients studied were those considered to provide the best compromise between the precision of SUV measurements on the one hand and the quality of images displayed with an SUV scale, on the other hand, which needs to be high enough to provide easy visual detection of areas of abnormal bone metabolism—i.e., high foci detectability, even for a 0.6-ml sphere volume and with significant SUVmax underestimation only for spheres <10 ml (Figure 1). The choice of such reconstruction parameters favoring contrast-to-noise ratio also had the advantage of minimizing the confusing influence of noise level on the determination of SUVmax (24, 25). SUVmax was additionally easier to use here than SUVmean in the absence of any precise knowledge regarding the limits of the diseased bone volumes.

It must be recognized that the lower spatial resolution of these SPECT images, in comparison with actual PET images, and the resultant higher partial volume effect, remains a limitation for SUV measurement. Despite this limitation, however, very consistent results were observed in our longitudinal study of patients with vertebral fractures and/or compressions, thereby reinforcing the potential usefulness of this method for SUV-based image scaling. These results include the stability over time of the SUVmax from intact control reference vertebrae. The T1 vertebrae were chosen for this stability analysis because of their very low risk of osteoporotic fractures (16) and because they were visually intact on the SPECT/CT images of all of our patients.

The results also show a dramatic decrease over time for the SUVmax from initially fractured vertebrae, as would be expected, although elevated SUVmax levels were still documented over a 7-month period (Figure 3). Such persistence of elevated SUVmax over the longer term is not surprising considering that bone scintigraphy may remain visually abnormal even at 1 year after a vertebral compression fracture (26).

An additional observation was that as many as 98% of the fractured vertebrae exhibited SUVmax >7.5 during the 7-month period following the acute episode, whereas this was highly unusual in the reference intact T1 vertebrae with only 4% exhibiting SUVmax >7.5. Therefore, it may be considered that image scaling favoring the identification of bone structures with a >7.5 SUV level would help to detect most bone fractures, even long after the acute traumatic episode.

**Figure 4:**
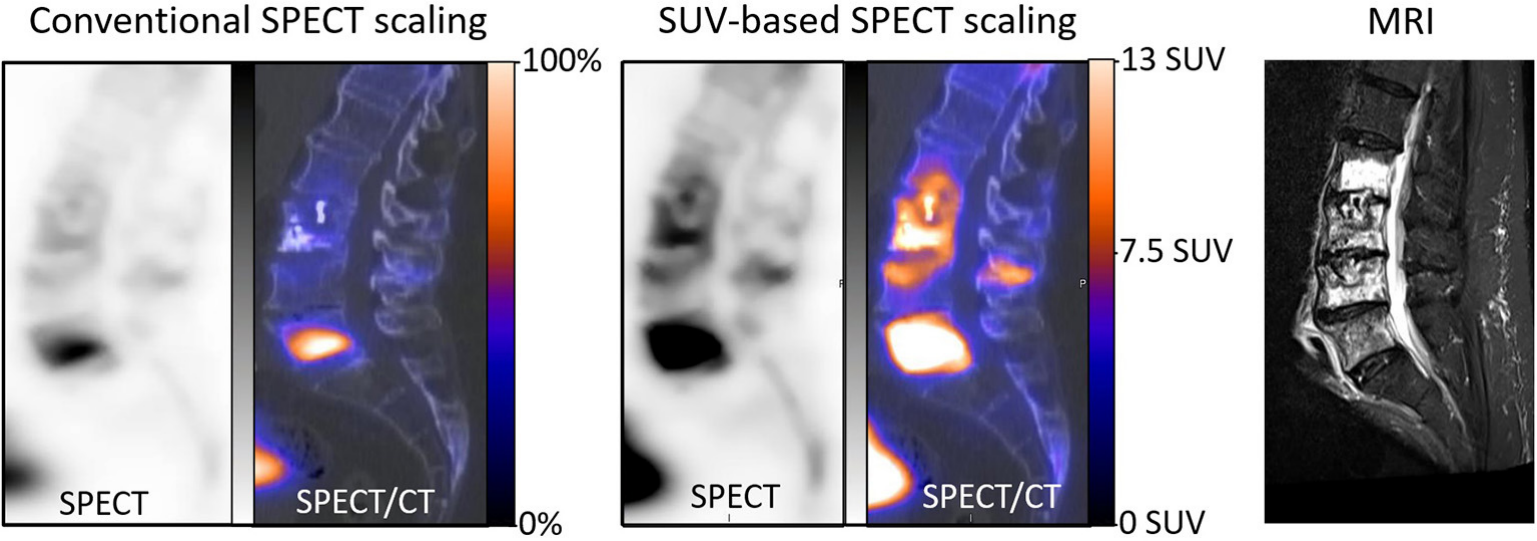
Sagittal SPECT/CT images and MRI centered on the lumbar spine and recorded in a 70-year-old man with a history of L3 cementoplasty, 2 months previously. This SPECT/CT recording had been planned for suspicion of new painful fractures. The SPECT images were initially displayed with a conventional scaling method according to the maximal voxel intensity and further visual adjustment, and a high uptake was observed mainly on the body of L5 (left), suggesting the potential benefit of an augmentation procedure targeted on L5. However, the physician in charge of the augmentation procedures found these images to be discordant, as compared with the lesions extending from L2 to L5 on an MRI recorded during the same period (right). In a second step, the scale of SPECT images was modified, with a maximum level set at 13 SUV, thus saturating the high uptake level from L5 and providing a clear identification of the bone structures reaching a level ≥7.5 (median). On these new SPECT images, much more diffuse abnormalities were detected, extending from L2 to L5 and with vertebral SUVmax ranging from 10.5 to 31.5, in accordance with the observations provided by MRI.

Until now, the scaling of bone SPECT image has routinely been done in an empirical and subjective visual way, mainly based on the % of maximal voxel activity. Standardized SUV-based scaling would likely enhance the reproducibility and robustness of bone SPECT analysis, mimicking what is currently achieved and recommended for oncologic PET imaging (1, 2). It might also alleviate the difficulties encountered when scaling images where bone lesions are very diffuse (i.e., a super scan) or having a very high maximal activity level. This point is illustrated in Figure 4 by the SPECT images from an actual clinical case where the diagnosis could be corrected with a secondary analysis, thanks to the application of our proposed SUV-based scaling method. In addition, the usefulness of fixed SUV scales could be particularly significant for longitudinal follow-up analysis, as illustrated in Figure 3 by the easy visual identification of bone areas with significant increased or decreased metabolism.

Lastly, recording times were only 12 to 15 min for a full-3D SPECT acquisition on the entire spine. This latter property is likely advantageous in patients for whom a prolonged supine position on the camera bed is difficult to endure due to their painful fractures.

In conclusion, bone scintigraphy of vertebral fractures may be obtained with this high-speed CZT-SPECT/CT system with fast 3D acquisitions and an image displayed using a reliable SUV scale, approaching what is commonly achieved and recommended for whole-body PET imaging.

## Data Availability Statement

The original contributions presented in the study are included in the article/Supplementary Material, further inquiries can be directed to the corresponding author/s.

## Ethics Statement

The study was approved on January 22, 2021 by the Ethics Committee of the CHRU of Nancy (reference number 297). This research complied with the principles of the Declaration of Helsinki. Informed consent was obtained from all individuals included in the study. The patients/participants provided their written informed consent to participate in this study.

## Author Contributions

ABa, P-YM, and LI: writing of the manuscript. ABa, AV, ABl, P-YM, and LI: revision of the manuscript. All authors contributed significantly to the analysis and interpretation of the data.

## Conflict of Interest

CHRU-Nancy, Department of Nuclear Medicine and Nancyclotep Imaging Platform is a site visit of the Veriton camera for Spectrum Dynamics. The authors declare that the research was conducted in the absence of any commercial or financial relationships that could be construed as a potential conflict of interest.

## Publisher's Note

All claims expressed in this article are solely those of the authors and do not necessarily represent those of their affiliated organizations, or those of the publisher, the editors and the reviewers. Any product that may be evaluated in this article, or claim that may be made by its manufacturer, is not guaranteed or endorsed by the publisher.

## Supplementary Material

Supplementary Figure Comparison of results provided by 2 recommended OSEM reconstruction processes, the first one favoring spatial resolution but requiring high count statistics (red lines) and the second one favoring contrast-to-noise ratio and finally selected for the present study (blue lines), with **(A)** the analyses of the evolutions of the maximal voxel activity concentration measured on the spheres of the IEC phantom and expressed relative to the actual activity concentration, **(B)** the contrast/noise ratio determined on the IEC phantom, and **(C)** representative tomographic SPECT slices from the IEC phantom and **(D)** a patient bone sagittal slice passing through a fractured T4 vertebra.
