# Supplementary figures and images for: Bone Scintigraphy of Vertebral Fractures With a Whole-Body CZT Camera in a PET-Like Utilization
Source: Front Nucl Med. 2021 Sep 10;1:740275. doi: 10.3389/fnume.2021.740275 (PMC11440846; doi:10.3389/fnume.2021.740275)

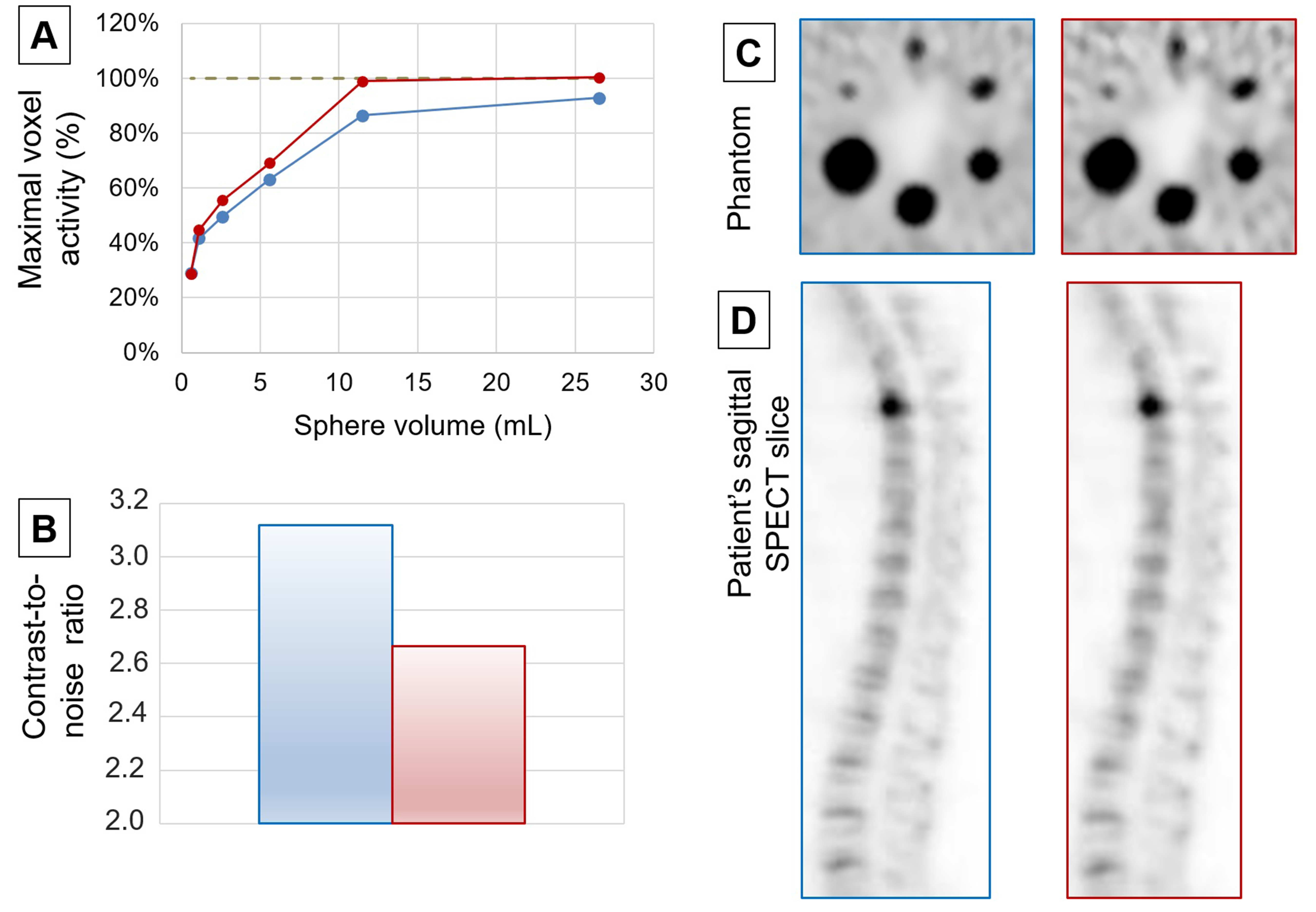

Supplement: Supplementary Figure — Comparison of results provided by 2 recommended OSEM reconstruction processes, the first one favoring spatial resolution but requiring high count statistics (red lines) and the second one favoring contrast-to-noise ratio and finally selected for the present study (blue lines), with (A) the analyses of the evolutions of the maximal voxel activity concentration measured on the spheres of the IEC phantom and expressed relative to the actual activity concentration, (B) the contrast/noise ratio determined on the IEC phantom, and (C) representative tomographic SPECT slices from the IEC phantom and (D) a patient bone sagittal slice passing through a fractured T4 vertebra. [file Image_1.TIFF]
